# Enabling Sensor-Integrated and Sustainable Aerospace Structures Through Additively Manufactured Aluminium Mechanisms for CubeSats

**DOI:** 10.3390/s26010281

**Published:** 2026-01-02

**Authors:** Bernardo Alves, Rafael Sousa, Ricardo Coelho, Daniel Gatões, Luís Cacho, Ricardo Branco, Vítor Miguel Santos, Patrícia Freitas Rodrigues

**Affiliations:** University of Coimbra, Centre of Mechanical Engineering, Materials and Processes (CEMMPRE-ARISE), Department of Mechanical Engineering, 3040-248 Coimbra, Portugal; bernardo.alves@uc.pt (B.A.); uc2020233770@student.uc.pt (R.S.); uc2020233708@student.uc.pt (R.C.); daniel.gatoes@uc.pt (D.G.); luis.cacho@dem.uc.pt (L.C.); ricardo.branco@dem.uc.pt (R.B.); pf.rodrigues@uc.pt (P.F.R.)

**Keywords:** CubeSats, metallic additive manufacturing, sensor mechanisms, space exploration

## Abstract

CubeSats are a fundamental tool of space exploration, allowing for the testing of novel ideas that can be upscaled to more efficient satellite systems. This work presents the development and characterisation of an additively manufactured aluminium mechanism designed to enable the self-functionalisation of CubeSat structures through material extrusion metal additive manufacturing, as a foundation for sensor integration. A space-grade AlSi7Mg alloy was selected and prepared as a filament to print a fully functional hinge geometry, aiming to evaluate the feasibility of producing movable metallic components using a low-cost and sustainable extrusion-based process. Produced parts were subjected to debinding and vacuum sintering, achieving a densification above 85% and an average hardness of 52.2 HV. Further characterisation, including micro-computed tomography, X-ray diffraction and dynamic mechanical analysis, was used to assess the microstructural integrity, present phase, and mechanical behaviour of the sintered components. The designed shrinkage-compensated hinge mechanism preserved its rotational mobility after sintering, validating the mechanical inter-locking strategy and the design for additive manufacturing methodology used. The results demonstrate that material extrusion enables the fabrication of lightweight, functional, and integrated aluminium mechanisms suitable for sensor incorporation and actuation in small satellite systems. This proof-of-concept highlights material extrusion as a sustainable and economically viable route for developing intelligent aero-space structures, paving the way for future adaptive and sensor-integrated CubeSat subsystems.

## 1. Introduction

The increasing demand for miniaturised, multifunctional and autonomous space systems has driven the development of lightweight, compact and adaptive satellite structures capable of performing multiple functions within strict mass, energy and volume constraints. Among these, CubeSats, standardised nanosatellites with 1U to 12U configurations, have become an affordable and efficient platform for in-orbit technology demonstration, Earth observation and communication missions, while significantly reducing development time and launch costs [1,2].

Initially conceived for academic purposes, CubeSats have evolved into versatile platforms for testing new materials, instrumentation (such as optics, detectors and sensors) and propulsion systems, contributing to the democratisation of space access [2]. Their growing mission complexity, which increasingly involves intersatellite communication, attitude control and propulsion, has created the need for components that integrate structural integrity, mechanical adaptability and sensing capability within extremely limited physical envelopes [3,4]. Furthermore, multifunctional satellite architectures, capable of combining mechanical strength, electrical conductivity and sensing performance, highlight the evolution from purely structural elements to intelligent and self-monitoring systems [3] as well as the importance of adopting advanced materials and fabrication strategies that enable functional integration from the design stage to the final production of CubeSat structures and deployables [5].

Recent advances in additive manufacturing (AM) have transformed the design and production of aerospace components, enabling the creation of complex and topology-optimised structures that were previously unfeasible through conventional manufacturing routes [6]. Techniques such as laser powder bed fusion, directed energy deposition and material extrusion (MEX) enable precise control of geometry and microstructure, providing new solutions for weight reduction, performance optimisation and design freedom in critical components [5,6]. In the specific context of CubeSat systems, recent studies demonstrate that additive manufacturing supports on-demand fabrication, part consolidation and mass minimisation, which are essential to improve payload efficiency and sustainability during launch and operation [7,8,9]. These advantages make AM a key enabler for the next generation of adaptive and multifunctional satellite structures.

Material extrusion, a filament-based process that combines extrusion, debinding and sintering, provides a sustainable and cost-effective alternative for producing metallic components with controlled geometry and reasonable mechanical performance [10]. This technique can be implemented using relatively simple equipment, with lower energy consumption and reduced material waste, making it suitable for distributed or educational fabrication environments [11,12,13]. This technology has been successfully applied to stainless steel, copper and aluminium alloys, producing parts with mechanical properties compatible with low to medium stress structural applications [13,14]. The method also offers a high degree of design flexibility, enabling the fabrication of internal channels, lightweight lattices, and hybrid structures that are difficult to achieve by powder bed fusion [15]. However, its use in the aerospace sector has so far been limited to preliminary studies, and the production of functional or movable mechanisms, such as deployable hinges, adaptive frames or sensor-integrated joints, remains largely unexplored [16,17].

Aluminium alloys are among the most commonly used materials in CubeSat structural components due to their excellent combination of high specific strength, low density and corrosion resistance [18]. These alloys offer a balanced compromise between mechanical performance and manufacturability, making them ideal for lightweight structures that must endure launch loads while maintaining thermal and dimensional stability in orbit. Recent studies have shown that additively manufactured AlSi7Mg exhibits favourable mechanical properties and microstructural uniformity, which can be further optimised through tailored post-processing strategies [19,20,21].

Integrating sensing or actuation capabilities directly within aluminium components not only enhances their functionality but also reduces the need for secondary assemblies or fasteners, reducing weight and interfaces that often represent critical failure points under vibration or thermal cycling. As a result, embedding functionality into the structural geometry improves overall system reliability and simplifies assembly, both of which are essential for small satellite platforms operating under severe mass and volume constraints [14,22].

The emerging concept of sensor-integrated and sensor-ready structures embeds or couples sensing elements within the material itself, enabling real-time monitoring, actuation, and self-diagnosis [20]. This approach enables the design of intelligent systems capable of responding to environmental stimuli or structural changes without the need for external instrumentation, supported by AM processing [23,24].

Additive manufacturing plays a central role in this evolution, as it allows the fabrication of multifunctional components with tailored internal architectures that can host sensors, conductive networks, or stimuli-responsive materials [25]. Metallic-MEX provides geometry freedom and enables mid-print integration of sensing elements, while the thermal and microstructural data generated can support AI-based models for predicting densification, defect evolution, and mechanical performance [26,27].

Although this concept has been extensively explored in polymeric and composite systems, its application to metallic structures remains relatively limited. Metals present unique challenges related to anisotropy, porosity, and shrinkage during post-processing, all of which affect the stability and performance of integrated sensors [28,29]. Recent research in metal additive manufacturing is beginning to address these issues through improved process control and hybrid fabrication approaches that combine structural and functional materials within a single build [30,31].

In this context, the present study investigates the potential of metal extrusion to fabricate a functional aluminium hinge mechanism, serving as a proof-of-concept for a future sensor-integrated structural element for CubeSat systems. Beyond demonstrating the feasibility of producing a movable metallic component, the hinge is intended as a platform for future integration of embedded sensing elements, enabling in situ monitoring and active response capabilities within small satellite structures. A second objective of this work is to generate high-quality microstructural, mechanical and geometric datasets that can support machine-learning approaches for predicting sintering behaviour, defect evolution and performance variability in metal–MEX components.

The hinge was manufactured using an AlSi7Mg filament and processed through solvent debinding followed by vacuum sintering, resulting in densification above 85%. The printed samples were evaluated for internal architecture, phase composition, thermo-mechanical response, and surface hardness using micro-computed tomography, X-ray diffraction, dynamic mechanical analysis, and microhardness testing. These characterisations verified the structural integrity and functional mobility of the component, demonstrating that material extrusion is a viable route for producing lightweight aluminium mechanisms that can accommodate future sensing integration in CubeSat applications.

## 2. Materials and Methods

**Figure 1:**
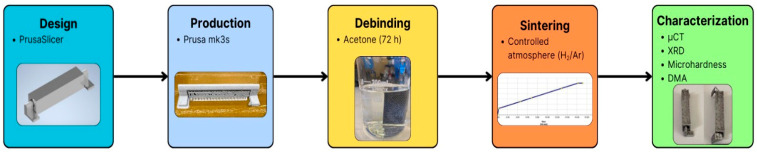
Experimental workflow of the study, including the main processing parameters for design, metal extrusion, debinding, sintering, and characterisation steps.

This study followed a four-step methodology: (i) numerical simulation for system optimisation and sensor integration in a 1U CubeSat structure, (ii) green-part fabrication via Material Extrusion with a functional insertion strategy, (iii) debinding and controlled sintering, and (iv) post-sintering characterisation including µCT, dimensional, microstructural and thermo-mechanical analyses (Figure 1). µCT and DMA were used as high-resolution diagnostic tools to characterise internal defects, interlayer quality and thermo-mechanical stability, generating datasets that can be leveraged for AI-driven modelling of sintering behaviour.

### 2.1. Materials

The AlSi7Mg filament with 60 wt.% developed at the University of Coimbra [14] was used. The feedstock followed established formulations for metal-MEX systems, combining gas-atomised AlSi7Mg powder (EN ISO 18273, purchased from Carpenter Additive, Philadelphia, PA, USA) with a multicomponent polymer binder system. After mixing and granulation, the feedstock was extruded into filament form and used directly in the fabrication of green parts.

### 2.2. Green-Part Manufacturing via MEX

Green components were fabricated using a Material Extrusion technique, with a Prusa MK3S-type printer (Prusa Research, Prague, Czech Republic), and the G-code was generated with PrusaSlicer software (version 2.9.3) using previously defined slicing parameters, as described by Alves B. et al. 2025 [14].

To maintain geometric stability during fabrication, established design-for-MEX practices were adopted. A brim was used to mitigate warping and improve build-plate adhesion, and snug-type supports were employed to stabilise local overhangs. Vertical holes and internal channels were modelled using teardrop-shaped profiles to minimise sagging in unsupported regions and preserve dimensional accuracy. This combination of design and process adjustments ensured the reproducibility and structural integrity of the green parts prior to thermal processing.

**Figure 2:**
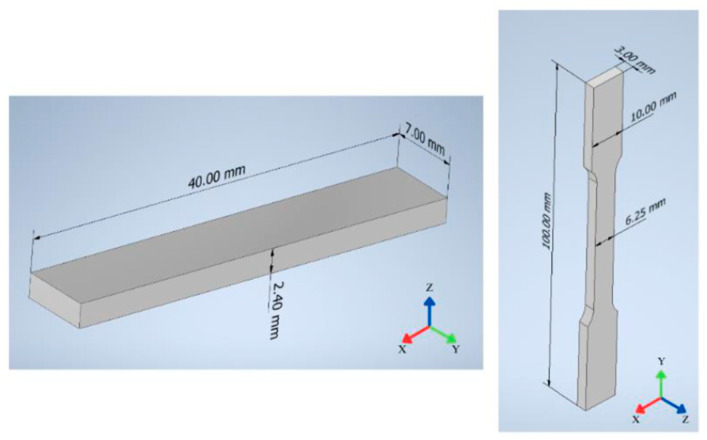
Geometries and dimensions of the printed specimens.

Two geometries were printed to enable the application of characterisation techniques to evaluate the properties of the aluminium alloy produced by this method, as shown in Figure 2. A third geometry was selected as a case study to incorporate a functional element. Additionally, to the previously described practices, the printing strategy incorporated a programmed mid-print pause, enabling the insertion of internal functional elements such as hinges, rotating links, or sensor housings. This procedure ensured that the functional element remained free to move after sintering while being fully encapsulated by the printed outer structure.

### 2.3. Debinding and Sintering

After fabrication, the components underwent a two-stage debinding and sintering cycle designed to remove the binder system and consolidate the metallic structure.

Debinding was performed through a solvent-assisted stage, submerged in an acetone solution for 72 h, which removed low-molecular-weight binder constituents and facilitated the formation of open porosity. This was followed by thermal debinding and sintering under controlled Ar/H_2_ (95/5%) atmosphere, during which the remaining polymeric components were gradually decomposed and volatilised under controlled heating. The temperature ramps were designed to avoid internal pressure build-ups and deformation of the fragile green structure. The debinding and sintering approach was implemented following the methodology presented by Alves B. et al. 2025 [14], allowing direct comparison with earlier results.

### 2.4. Post-Sintering Characterisation

A comprehensive set of techniques was used to evaluate the densification, structural integrity, and functional performance of the sintered components.

#### 2.4.1. X-Ray Microcomputed Tomography

X-ray microcomputed tomography (µCT) was used to quantify internal porosity, detect residual defects, and verify geometric fidelity. Reconstructed 3D datasets enabled inspection of pore morphology, distribution and potential delamination, while also confirming that the embedded mechanism remained unconstrained after consolidation. The equipment used for this analysis was the Bruker Skyscan 1275 (Bruker, Kontich, Belgium), located at IPN. Scans were performed at 80 kV and 125 μA, using a 1 mm aluminium filter and the step-and-shoot acquisition mode. The pixel size was set to 10 μm, and random movement mode was used. Reconstruction was performed on Bruker proprietary software (NRecon 1.7.4.6).

#### 2.4.2. Dimensional and Shrinkage Evaluation

Dimensional changes between green and sintered parts were assessed through direct measurements and µCT-derived models. Linear and volumetric shrinkage were quantified to evaluate densification of uniformity and potential anisotropic behaviour related to print orientation or part geometry.

#### 2.4.3. Structural Evaluation

X-ray diffraction was used to identify phases present after sintering and evaluate structural evolution. X-ray diffraction (XRD) was used to identify crystalline phases in the sintered specimens. Measurements were performed on a Philips X’Pert diffractometer (Philips, Egham, UK) at 40 kV and 35 mA, in Bragg–Brentano (θ–2θ) geometry with a cobalt anode (Co Kα1 = 0.178897 nm; Kα2 = 0.179285 nm). Scans covered 30–100° (2θ) with a 0.025° step and 1 s per step, enabling phase identification across processing stages.

#### 2.4.4. Hardness and Thermo-Mechanical Testing

Vickers’ microhardness measurements were performed to evaluate the average hardness along the parts produced by the MEX process and to analyse the influence of this manufacturing method on the material’s local mechanical properties. The tests were carried out using a Duramin Digital Vickers hardness tester (Struers A/S, Ballerup, Denmark). A load of 490 mN and a dwell time of 15 s were applied for all measurements.

Dynamic Mechanical Analysis (DMA) was used to characterise storage modulus and damping behaviour over a relevant temperature range, providing insight into the thermo-mechanical response of the sintered alloy. DMA was performed on a Triton Tritec 2000 to evaluate storage modulus and temperature dependence, enabling assessment of changes in mechanical behaviour over the selected temperature range. The analysis was performed on dual cantilever bending mode with a fixed displacement of 0.01 mm, using three different frequencies (1, 5 and 10 Hz). Five temperature cycles between −100 and 50 °C were performed at each frequency on a single sample, with the equipment capable of measuring only during heating.

### 2.5. Case Study: Fabrication and Functional Demonstration of an Integrated Mechanism

A case study was conducted to validate the ability of MEX to produce sintered metallic structures containing fully functional embedded mechanisms representative of deployable CubeSat components.

#### 2.5.1. Printing Methodology

The internal and external components were modelled independently. The rotating inner element was designed with clearances to accommodate sintering shrinkage, while the outer shell was modelled to encapsulate it without restricting movement. Geometric tolerances, rotational axes, and interfaces were defined during this stage.

The assembly was imported into slicing software, where build orientation, layer height, infill density, printing temperature and supports were defined. A programmed pause layer was added to allow insertion of the internal component.

**Figure 3:**
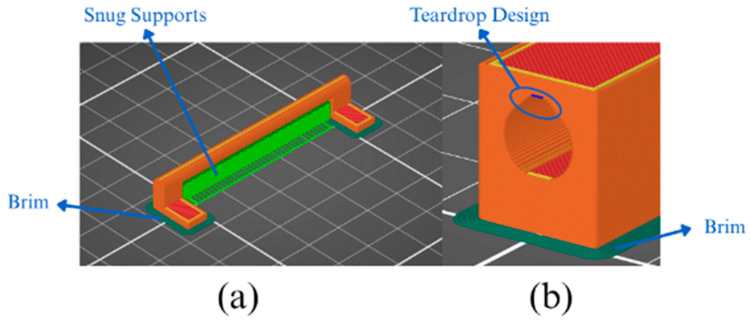
(**a**) Inner supports and brim that stabilise the rotation axis in the inner hinge; (**b**) Teardrop-shaped opening and external brim that accommodate the rotational movement of the mechanism.

In parallel with the choice of printing strategy, several process parameters were adjusted to improve part quality. A brim was added around the base of the parts to reduce warping, an effect particularly relevant for long geometries such as the present hinge. Snug-type supports were used to form overhanging regions, especially within the inner hinge, which contains surfaces printed in mid-air. In addition, a vertical hole in the outer hinge was designed with a teardrop-shaped cross-section, a common additive manufacturing solution that prevents material sagging at the unsupported upper section of the aperture. These features and printing parameters are shown in Figure 3.

The internal component was printed first and temporarily stored. The external shell was printed up to the pause layer, after which the printer halted automatically. The internal mechanism was manually positioned and aligned, and printing resumed, encapsulating it within the structure.

#### 2.5.2. Debinding and Sintering of the Assembly

The assembled green component underwent debinding and sintering using the same thermal cycle described in Section 2.3. The profile was selected to achieve densification while preventing metallurgical bonding between the moving element and the surrounding matrix.

#### 2.5.3. Functional Verification

After sintering, the mechanism was actuated to confirm rotational freedom, interface integrity, and mechanical robustness. This procedure demonstrated the feasibility of producing deployable, functional mechanisms using metal-MEX followed by debinding and sintering.

## 3. Results

The specimens were characterised to evaluate their suitability for structural and sensing-oriented applications in CubeSat assemblies. Emphasis was placed on defect morphology, dimensional evolution, and thermo-mechanical stability, as these factors directly influence the potential integration of embedded sensors and the generation of datasets for data-driven models in smart manufacturing.

### 3.1. Green and Sintered States

**Figure 4:**
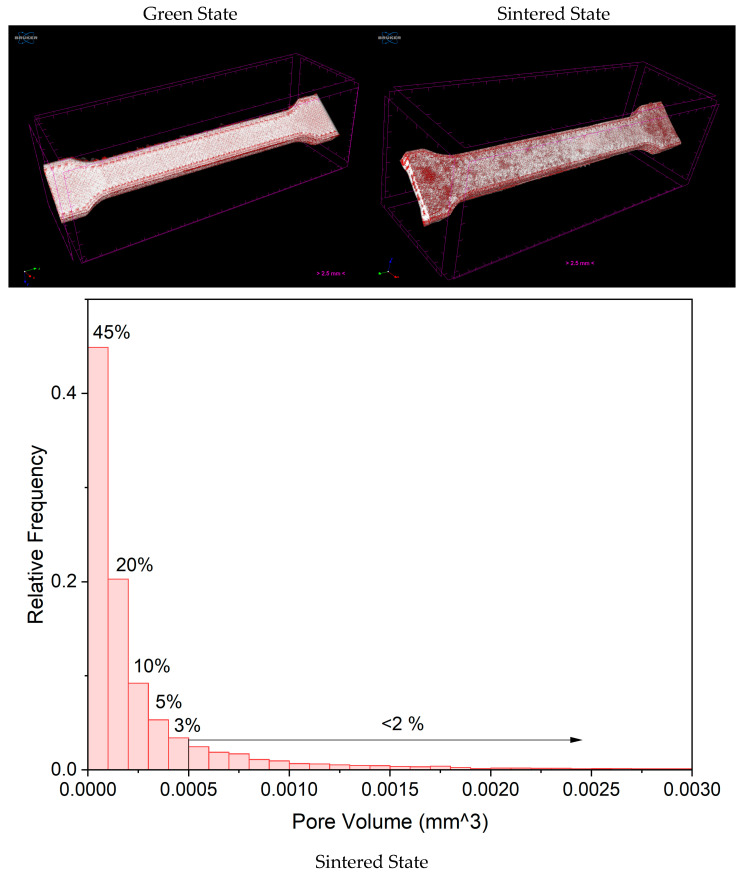
CT image of the green and sintered specimens showing printing-related defects, porosity and a distribution of pore volume.

The µCT analysis of the green specimens revealed internal porosity primarily aligned along the infill paths, together with small gaps at the interfaces between the perimeter and infill regions (Figure 4). These defects are characteristic of filament-based metal extrusion and originate from incomplete fusion between successive extruded filaments, minor fluctuations in material flow, and insufficient overlap during deposition [32]. Although typical of MEX, such imperfections are critical because they influence defect evolution during debinding and sintering, where small voids may close through diffusion or, conversely, expand into larger discontinuities if binder removal is not fully uniform.

After sintering, the parts exhibited a marked dimensional contraction, corresponding to an overall volumetric shrinkage of approximately thirty percent (Table 1). Shrinkage in the X and Y directions was almost isotropic, whereas a more pronounced reduction of around eighteen percent was observed along the Z direction, consistent with the intrinsic anisotropy of the layer-by-layer process [21,33]. The low standard deviation across all samples indicates that, despite substantial shrinkage, the process is highly repeatable, which is essential for defining functional tolerances in mechanisms or sensor housings. Table 1 summarises this dimensional evolution and provides a basis for predicting the final geometry of MEX components.

The µCT evaluation of the sintered parts revealed several critical defects. Surface blistering was observed, most likely due to incomplete binder removal and localised interaction with the support crucible. Internal voids and delamination zones were also detected, particularly in regions already identified as weak in the green state. These defects confirm that diffusion and particle bonding were insufficient during consolidation and highlight the need to optimise both the thermal debinding profile and the initial filament bonding quality. The comparison between green and sintered states (Figure 4) clearly shows that small inter-filament gaps can transform into structurally significant defects during densification. Furthermore, the pore size distribution clearly shows a prevalence of pores with less than 100,000 µm^3^, suggesting that the greater number of pores originates from the sintering step, and aligns with the measured relative density.

### 3.2. XRD-Based Analysis of Structural Changes After Sintering

**Figure 5:**
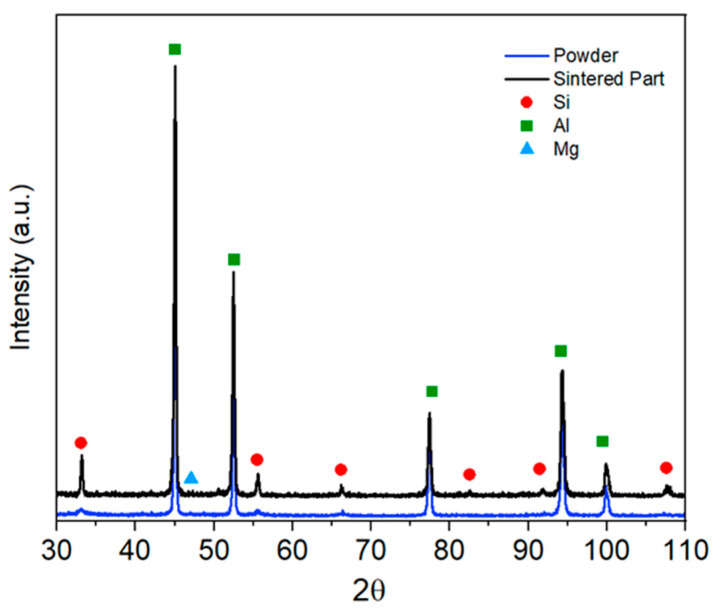
XRD patterns of aluminium alloy powder and the sintered part.

The X-ray diffraction analysis confirmed that both the AlSi7Mg feedstock powder and the sintered components retained the characteristic Al and Si phases expected for this alloy system, as shown in Figure 5. The absence of additional diffraction peaks indicates that oxidation during sintering was effectively suppressed and that no detrimental secondary phases formed. This is a relevant observation for aluminium-based MEX parts, where oxygen uptake can impair densification, reduce mechanical integrity, and compromise the stability of structures intended to host embedded sensors.

The main change observed after sintering was a noticeable reduction in the intensity of the Mg peaks. This attenuation suggests partial volatilisation of magnesium, consistent with its high vapour pressure at temperatures near 600 °C [34]. Magnesium is the most volatile constituent in Al–Si–Mg alloys, and its depletion during high-temperature processing is well established. Although the primary phases remained unchanged, the reduction in Mg content may influence the alloy’s mechanical response, since magnesium contributes to solid-solution strengthening and precipitation-hardening mechanisms [35].

A mechanistic interpretation of these observations can be obtained by considering the potential reactions between Mg and the alumina film surrounding the powder particles:

3Mg + 4Al_2_O_3_ → 3Al_2_MgO_4_ + 2Al

3Mg + Al_2_O_3_ → 3MgO + 2Al

These reactions show that magnesium can disrupt the Al_2_O_3_ passivation layer during the early stages of sintering [36]. This disruption is beneficial for promoting metal-to-metal bonding, since it weakens the otherwise stable oxide shell. However, it also accelerates Mg volatilisation, contributing to the reduced peak intensity detected by XRD in the sintered specimens. The dual effect of Mg, facilitating oxide breakdown while being lost from the system, helps explain the observed microstructural evolution and the mechanical performance trends discussed in later sections.

### 3.3. Mechanical Behaviour

A comparison with literature shows that the hardness of AlSi7Mg produced in this study is lower than values typically reported for laser-based additive manufacturing. AlSi7Mg fabricated by Selective Laser Melting often reaches hardness levels close to 105 HV [37], whereas the MEX-derived and sintered specimens examined here achieved an average of 52.2 HV. Although this difference is significant, it is important to emphasise that hardness in aluminium alloys is strongly dependent on densification behaviour and thermal processing history. These factors often have a greater influence on mechanical performance than the nominal alloy composition alone.

This interpretation is supported by previous work on a 7000-series aluminium alloy processed using the same MEX and sintering route, where a maximum hardness of 65.4 HV was achieved at 600 °C [38], only moderately higher than those obtained in the present study.

These results indicate that the mechanical response of MEX-produced aluminium alloys is governed primarily by porosity levels, particle bonding, and elemental retention during sintering, rather than by differences between alloy series. The relatively low hardness measured here is consistent with the XRD findings, which indicate partial loss of silicon and magnesium during the thermal cycle. Both elements play key roles in strengthening Al alloys; silicon contributes to load-bearing eutectic phases, while magnesium enhances solid-solution and precipitation hardening. Their depletion, therefore, provides a direct explanation for the reduced hardness and highlights the need to optimise sintering conditions to minimise volatilisation.

### 3.4. Dynamic Mechanical Behaviour

**Figure 6:**
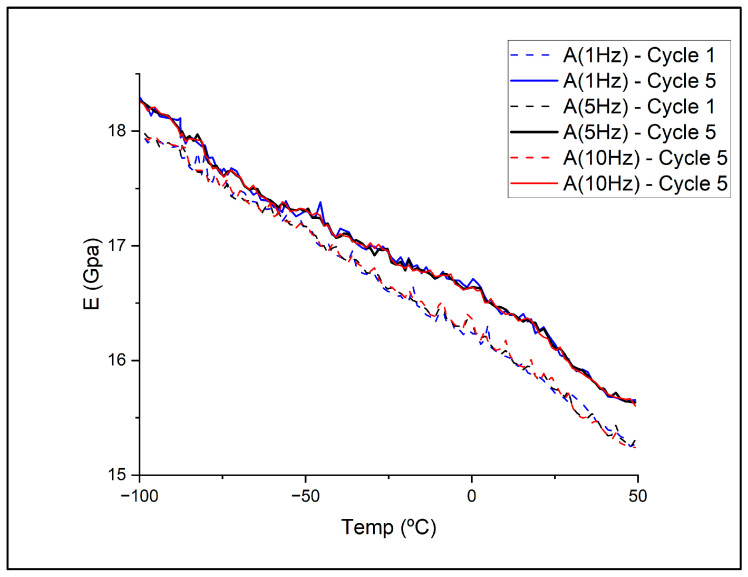
Storage modulus variation with temperature at 1, 5, and 10 Hz.

Dynamic Mechanical Analysis was performed to characterise the thermo-mechanical response of the sintered alloy under cyclic loading. Figure 6 shows the evolution of the storage modulus as a function of temperature for three excitation frequencies (1, 5 and 10 Hz), measured during the first and fifth cycles. In all cases, the modulus decreases progressively with increasing temperature, reflecting the expected thermal softening of aluminium as atomic mobility increases.

The curves for the first and fifth cycles trail closely, indicating that repeated thermal and mechanical loading did not introduce measurable degradation within the investigated range. This response suggests that the microstructure remains mechanically stable during cycling, with no evidence of softening, cumulative damage, or irreversible changes. Minor frequency-dependent variations fall within the typical scatter of DMA experiments. The slight increase in modulus observed in the fifth cycle is attributed to stabilisation of the specimen-clamp interface and relaxation of residual stresses, effects frequently reported in metallic DMA studies.

### 3.5. Case Study Analysis

#### 3.5.1. Three-Dimensional Model

The full CubeSat structure was modelled, but only one specific feature was selected for fabrication: the hinge mechanism located on a lateral panel, which also incorporates the top face of the satellite. This element enables the controlled opening of the structure to allow the integration of internal subsystems. It is also the only feature of the frame that cannot be produced using conventional subtractive manufacturing due to its enclosed rotational interface.

**Figure 7:**
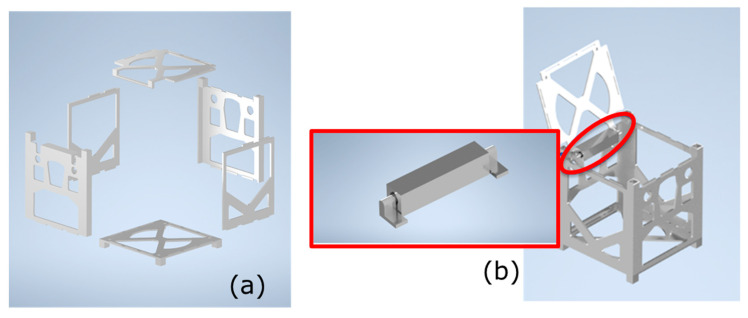
(**a**) CubeSat structural assembly exploded view and (**b**) Open CubeSat configuration with hinge mechanism highlighted.

Although the remaining CubeSat elements were not fabricated, they were designed to be manufacturable without support structures. For this study, CAD modelling focused exclusively on the hinge system (Figure 7), shown both as an isolated assembly and in its intended position on the CubeSat frame.

Beyond enabling deployment, this hinge was selected because it represents a promising site for direct integration of sensing elements. Its geometry naturally accommodates position sensors, vibration sensors or embedded micro-accelerometers that could provide structural health monitoring and in-orbit diagnostics. By demonstrating that the hinge can be printed as a functional mechanism, the present study establishes a foundation for future work on embedding sensors in metallic CubeSat components.

#### 3.5.2. Slicing and Parameterisation

Once the CAD model was completed, the geometry was imported into the slicing software to generate the G-code required for fabrication. Two printing strategies were initially evaluated. The first involved producing the entire hinge assembly in a single operation. Although this approach is conceptually straightforward, it requires internal support structures to print the inner hinge surfaces. In practice, the metallic filament prevented the support from forming correctly, resulting in deposition defects that either prevented successful completion of the print or compromised subsequent processing steps.

A second strategy was therefore adopted. In this method, the inner hinge component was printed separately and later inserted during the fabrication of the outer hinge. This mid-print insertion approach eliminated the need for internal supports between the two components. It also prevented any support residue from remaining in the narrow clearances of the mechanism, which would have been extremely difficult to remove due to the tight tolerances required for the hinge to function. This solution demonstrates a particular advantage of the MEX process, since the ability to pause printing and insert an internal component is not feasible in most other additive manufacturing techniques.

**Figure 8:**
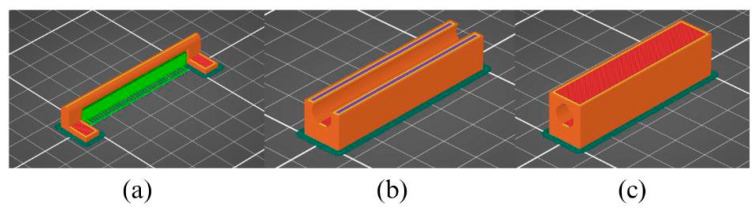
(**a**) Slicing view of the inner hinge; (**b**) slicing view of the outer hinge at the programmed pause layer; (**c**) slicing view of the outer hinge at the final layer.

With the strategy defined, fabrication proceeded in two stages. The inner hinge was printed in its entirety first. The outer hinge was then printed with a programmed pause at a predetermined layer height. This layer was selected to ensure that the printer nozzle would not collide with the inserted component when printing resumed. Figure 8 illustrates the stages involved in implementing this approach.

#### 3.5.3. Printing of the Integrated Mechanism

**Figure 9:**
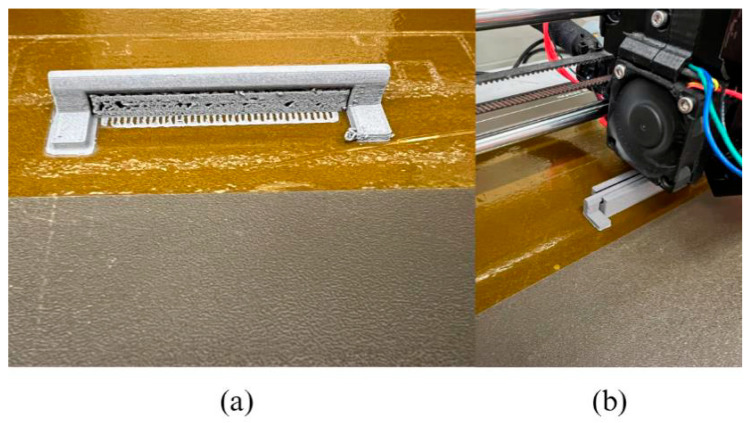
Printed strategy: (**a**) inner hinge; (**b**) outer hinge.

The hinge mechanism was fabricated using the previously defined mid-print insertion strategy. Figure 9a shows the inner hinge immediately after printing, still attached to the temporary supports that ensured the correct formation of its overhanging features. These supports were manually removed with care to preserve the dimensional tolerances required for assembly. The outer hinge was then printed and paused at the designated layer height to allow the insertion of the inner component, as illustrated in Figure 9b. Printing resumed without collision, completing the encapsulation of the mechanism.

This approach enabled the successful fabrication of the full green assembly while maintaining the clearance necessary for post-sintering movement.

#### 3.5.4. Debinding, Sintering and Functional Verification

**Figure 10:**
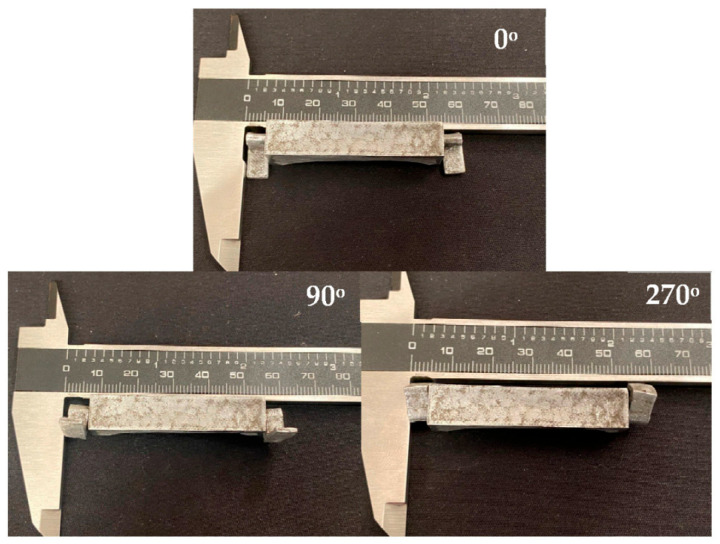
Final printed CubeSat hinge rotated by: 0°, 90° and 270°.

The assembled hinge underwent the defined debinding and vacuum sintering cycle, followed by light sanding to remove the superficial alumina layer. After processing, the final component, shown in Figure 10 (0°), preserved the mechanical independence of the internal and external elements. This confirms that no unintended bonding occurred during consolidation. The rotational motion remained fully functional after sintering, as illustrated by the different positions in Figure 10 (90 and 270°) and in the Supplementary Materials (Video S1—functional verification), demonstrating that the dimensional clearances defined in the green state were successfully maintained through densification.

These results confirm that metal MEX, when combined with a mid-print insertion strategy, can produce compact metallic mechanisms that remain operational after thermal consolidation. The hinge also constitutes a promising platform for integrated sensing because its internal rotational channel provides a naturally protected region that can accommodate position sensors for deployment monitoring, vibration or acoustic sensors for structural health assessment, or microaccelerometers for evaluating CubeSat microvibrations and attitude perturbations. The ability to fabricate the mechanism as a sealed structure while keeping internal motion unaffected by sintering enables the integration of sensing elements without the need for adhesives, fasteners or post-assembly steps, which is particularly advantageous in space applications where reliability and mass efficiency are critical.

The digital and experimental data generated in this case study, including geometric evolution, shrinkage behaviour, defect mapping, and thermo-mechanical response, also represent a multimodal dataset capable of supporting AI-assisted analysis. These datasets can contribute to the prediction of densification behaviour, defect evolution, and the expected mechanical response of sensor-integrated MEX components. Therefore, the hinge serves not only as a demonstration of manufacturability but also as a foundational element for advancing intelligent and data-driven CubeSat structures.

## 4. Discussion

The results presented in this work demonstrate that metal Material Extrusion is capable of producing functional aluminium mechanisms with geometric fidelity and mobility preserved after sintering. This establishes a technical foundation for the use of MEX in CubeSat subsystems. At the same time, several microstructural, dimensional, and chemical features observed throughout the study emphasise the need to integrate sensing and data-driven methodologies to advance MEX toward the smart reliable manufacturing of aerospace components.

The green-state analysis revealed that inter-filament gaps and porosity aligned along extrusion paths are inherent to the deposition process. These features were shown to evolve into larger voids or local delamination after sintering, demonstrating that the initial defect distribution strongly influences consolidation. The µCT datasets obtained here capture this evolution with high spatial resolution and therefore constitute an important resource for machine learning models aimed at predicting defect growth or identifying failure-prone regions based on the green-state geometry. Integrating µCT-derived defect maps with convolutional or graph-based neural networks could enable real-time print-quality assessment or provide corrective feedback for future builds, both key elements of smart manufacturing pipelines.

Dimensional analysis revealed stable shrinkage behaviour, with approximately thirty percent volumetric contraction and nearly isotropic behaviour in the X and Y directions. The more pronounced reduction along the Z axis reflects the intrinsic anisotropy of layer-wise processing. Although this shrinkage is predictable and repeatable, it introduces challenges for components with tight tolerances, especially mechanisms and sensor housings. AI-assisted shrinkage compensation models trained on datasets such as those presented here could support automatic adjustment of CAD geometries or toolpaths, reducing the need for empirical tuning. This strategy has the potential to greatly improve dimensional accuracy and reduce scrap rates, which is essential in distributed manufacturing environments where MEX systems may not operate under identical conditions [39].

The XRD analysis indicated that the alloy retained its primary Al and Si phases, with a reduction in Mg content after sintering. This volatilisation behaviour is consistent with the known thermodynamics of Mg at elevated temperatures and may contribute to the lower hardness observed in the sintered parts. Incorporating XRD data into predictive models may help anticipate the extent of elemental loss under different sintering atmospheres or cycle profiles, enabling future optimisation through data-driven thermal process design.

Mechanical characterisation further supported the combined effects of porosity, shrinkage, and Mg loss. The hardness values, although lower than those reported for laser-based AM techniques, are consistent with expectations for aluminium alloys processed through MEX. DMA results confirmed that the thermo-mechanical response remains stable under cyclic loading, with predictable stiffness reduction as temperature increases and no indication of structural degradation. This behaviour is favourable for CubeSat components exposed to repeated thermal transitions during orbit and is also important for sensor integration, since stable modulus profiles improve the reproducibility of strain or vibration signals. However, the values of elastic moduli obtained from DMA heavily suggest a need for further processing optimisation, specifically during debinding and sintering, as supported by green to sintered porosity distribution obtained via µCT.

From a structural–functional perspective, the microstructural heterogeneity inherent to metallic MEX presents both challenges and opportunities for integrating sensing technologies into CubeSat components. Local gradients in porosity, bonding density, and phase composition can produce variations in elastic modulus and damping behaviour that are not captured by bulk measurements but may strongly influence sensor response at the microscale. Establishing quantitative correlations between these local mechanical features and sensor signal fidelity will be essential to define design thresholds for embedded sensing architectures. Moreover, coupling in situ sensing data with process monitoring and machine learning could enable adaptive printing strategies in which deposition parameters are dynamically adjusted to maintain the stiffness and integrity required for reliable sensor–structure interaction. In this context, MEX not only provides the manufacturing pathway for multifunctional components but also the experimental platform for developing closed-loop, self-calibrating systems capable of autonomous performance validation in orbit.

The case study demonstrated one of the most distinctive advantages of MEX compared to other metal AM processes: the ability to interrupt a build, insert an internal element, and resume printing. This mid-print insertion strategy enabled the hinge mechanism to be fabricated as a single, fully enclosed assembly while preserving post-sintering mobility. Beyond enabling deployable structures, this characteristic opens a direct route to embedding sensors during printing, such as temperature sensors, position sensors, or vibration sensors, without requiring fasteners, adhesives or post-processing assembly steps. Some examples include temperature sensor via integrated shape memory alloys, enabling critical temperature identification via phase change, with the added possibility of acting directly as an actuator [40]; attitude control via embedded coils [41]; or even strain sensors via piezoelectric patches [42].

From a broader manufacturing perspective, metal MEX naturally aligns with smart and distributed fabrication principles by generating rich, multimodal datasets, spanning µCT, XRD, DMA, and hardness measurements, that serve as a foundation for AI-driven optimisation. These diverse data streams provide the foundations necessary for complex predictive modelling: µCT defect maps and dimensional analysis enable shrinkage and failure forecasting; XRD data regarding phase distribution and Mg depletion inform models on alloy stability and volatilisation; and thermo-mechanical profiles from DMA and hardness testing allow for predictions regarding fatigue, stiffness, and stability under thermal cycling. By correlating these microstructural and compositional indicators with green-state geometry, this integrated workflow not only facilitates the optimisation of densification and processing parameters but also establishes a pathway toward intelligent, sensor-integrated CubeSat structures capable of self-monitoring their own health, deployment, and vibration response.

The results of this study highlight not only the manufacturability of a functional metallic hinge but also its relevance as a building block for future AI-enabled, sensor-integrated spacecraft architectures. The combination of metal MEX, mid-print insertion, and data-driven process optimisation offers a promising direction for producing adaptive, lightweight, and intelligent components for next-generation CubeSat systems.

## 5. Conclusions

This study demonstrated that metal Material Extrusion can be used to fabricate functional aluminium mechanisms with sufficient dimensional stability and mobility to be considered for CubeSat structures. The successful production of a fully operational hinge, manufactured as an integrated assembly through a mid-print insertion strategy, confirms that MEX is capable of delivering compact mechanical components that remain functional after debinding and sintering. Nonetheless, optimisation of these two final processing steps is required to reduce porosity and enhance mechanical properties, along with fatigue testing with thermal cycling to support system functionality over the mission lifespan.

The results also highlight the potential of this manufacturing route to support future integration of embedded sensors, enabling new opportunities for health monitoring, deployment verification and structural diagnostics in small satellites. Moreover, the specific case of NiTi stands out as a viable candidate for embedded sensors due to its ability to retain shape memory effect through the thermal treatment required for MEX processing [14].

From a data standpoint, the experimental outputs collected throughout this work, including µCT defect maps, XRD phase evolution, DMA thermo-mechanical curves and shrinkage behaviour, constitute a rich multimodal dataset. These datasets can be used to train AI models to predict densification patterns, identify defect-prone regions, compensate for shrinkage, and improve overall process reliability. Such data-driven approaches align naturally with emerging smart manufacturing frameworks.

Taken together, the findings demonstrate that metal MEX is not only a feasible method for producing functional mechanisms but also a promising platform for the development of intelligent CubeSat structures. Its compatibility with mid-print encapsulation, its digital nature and the data it generates position this technology as a valuable contributor to future spacecraft designed with integrated sensing, autonomous behaviour and enhanced structural monitoring capabilities.

## Figures and Tables

**Table 1:** Dimensional Shrinkage Between Green and Sintered States.

Specimen	3D Model	Green State	Sintered State	Shrinkage (%)	Standard Deviation (%)
X (mm)	20.0	19.9	18.5	7.1	0.2
Y (mm)	15.0	15.0	13.8	7.8	0.2
Z (mm)	3.0	3.0	2.5	17.9	0.1
Vol. (mm^3^)	900.0	891.5	627.0	29.6	0.2

## Data Availability

The original contributions presented in this study are included in the article/Supplementary Materials. Further inquiries can be directed to the corresponding author.

## Supplementary Materials

The following supporting information can be downloaded at: https://www.mdpi.com/article/10.3390/s26010281/s1, Video S1: Functional verification.
